# Food hygiene, public health education and citizenship in Britain, 1948–1967

**DOI:** 10.1136/medhum-2020-012120

**Published:** 2021-09-03

**Authors:** Alex Mold

**Affiliations:** London School of Hygiene and Tropical Medicine Faculty of Public Health and Policy, London, UK

**Keywords:** history, public health, medical humanities

## Abstract

This article examines food hygiene campaigns in Britain between 1948 and 1967, using these as a way to explore the making of health citizenship and the relationship between state and citizen. The projection of hygienic citizenship amalgamated old concerns around morality, modernity and cleanliness, as well as new issues surrounding the changing position of women, the home and the rise of consumerism. Other ways of thinking about citizenship, such as social citizenship and consumer citizenship, were incorporated within food hygiene campaigns. The success or otherwise of such efforts points to a complex re-working of the connections between public health and its publics.

Notions of hygiene have long been central to practices designed to protect public health. The removal of waste, provision of clean water and food, prevention of the spread of infection and personal cleanliness are all important hygienic practices intended to keep individuals and communities well. The promotion of personal and public hygiene were crucial elements of the public health systems that developed in the West during the 19th and early 20th centuries. Good hygiene was often equated with good citizenship, as hygienic citizens strove to keep themselves and their communities well. Such practices did not go away even as the threat from infectious disease appeared to recede in the 20th century. Indeed, as the COVID-19 pandemic has demonstrated, hygiene remains strongly connected to notions of both health and citizenship. Good personal hygiene, and especially handwashing, was mobilised as the first line of defence against contracting and spreading COVID-19. Early attempts in Britain to halt the epidemic relied heavily on getting citizens to wash their hands thoroughly for 20 s. Although some of these efforts, such as the repeated sanitation of surfaces in public spaces has subsequently been shown to be of limited value in reducing the spread of an airborne disease, the importance of what has been branded ‘hygiene theatre’ cannot be denied (Thompson 2020).

The reasons for the persistence of hygienic thinking are multifaceted. One way in which we might account for this is by looking at how ideas of hygiene and hygienic citizenship were reformulated in the middle of the 20th century. The promotion of good food hygiene to prevent food poisoning was a major preoccupation of public health officials at the national and local level in Britain from the establishment of the National Health Service (NHS) in 1948 through to the late 1960s. As Anne Hardy has noted in her history of food poisoning the condition is ‘generally regarded as little more than an unpleasant and inconvenient episode’ despite it being a considerable public health problem throughout the world (Hardy 2015). Food poisoning was, and continues to be, a costly public health hazard with wider implications for food production, agriculture and biomedical research. In 1953, recorded incidences of food poisoning in England and Wales rose to 5277 cases compared with 3519 in 1952. Indeed, cases of food poisoning continued to increase reaching a peak of 8961 in 1955 and were still numbering in excess of 4000 by the mid-1960s (Ministry of Health 1965a). Efforts to prevent food poisoning may not always have been successful, but that does not mean that they were without significance. In this article, I will argue that the creation and communication of public health messages around food hygiene were strategy to reduce food poisoning, and a place where the re-constitution of notions of public health and citizenship in postwar Britain can be charted.

The period from the end of the World War II to the late 1960s saw significant change in the principal challenges to the public’s health as well as the design and delivery of public health services. Rates of infectious disease, long the scourge of public health, declined but incidences of chronic or non-communicable diseases crept upwards. The linking of many of these conditions (such as certain forms of cancer and heart disease) to individual behaviour (such as smoking, drinking, diet and exercise) required a shift in the orientation of public health policies and practices. This, in turn, had an impact on the relationship between public health and its publics (Mold et al. 2019). Existing models of what it meant to be a good citizen in relation to health needed to be adapted to address the new threats to the public’s health and to take into account broader shifts in the relationship between state and citizen.

Publicity to promote good food hygiene offers an excellent forum within which to observe such change because it combined both old fears and new approaches. ‘Hygienic citizenship’ had long been something all citizens (although not equally) were expected to strive for (Bashford 2003; Anderson 2006). In this article, I will suggest that hygienic citizenship did not disappear with the decline of infectious disease or the creation of the NHS. Rather, it was adapted to incorporate new ideas about what it meant to be a good citizen, encompassing aspects of social and consumer citizenship, while retaining some of the moral and gendered features commonly associated with hygienic citizenship. This is important for our understanding of the relationship between health and citizenship, and for thinking about citizenship as more than a set of legal or political rights and responsibilities (Grant 2016). To explore this dynamic, this article will begin by examining the historical relationship between hygiene, health and citizenship. It will then move on to provide an overview of the regulation of food hygiene from 1948 to 1967, as well assessing why it was that food poisoning, and efforts to prevent this through health education, were of concern. This was a time when other threats to public health, like smoking, appeared to be acquiring greater significance. Why was so much effort put into combating what might seem like a minor issue? Examining a selection of the campaign material produced, including public health information films, posters and codes of conduct reveals a set of deeper concerns. These revolved around older, longer-running issues connected to morality, modernity and cleanliness, as well as newer uncertainties about the changing position of women, and the rise of consumerism. Looking in more detail at who was targeted by food hygiene campaigns points to gender, class and ethnic distinctions in who was thought to be responsible for ensuring good food hygiene, and who was to blame when things went awry. Finally, considering the kinds of behaviours the campaign material was supposed to encourage, and the extent to which this was or could be realised, points to a complex re-working of the relationship between public health and its publics, and between state and citizen.

## Patient and public involvement statement

Patients and the public were not involved in the research for this article. This is a piece of historical research based on published sources, archival material, visual and audio-visual materials. Capturing the views of patients and the public in the past is not possible and asking current patients or members of the public about their views on and of such material would not be appropriate for a historical study.

## Hygiene, health and citizenship

The relationship between hygiene, health and citizenship is long-running and dynamic. In the interwar period, the influential Chief Medical Officer George Newman advocated an understanding of citizenship that combined individual and communal hygienic rights and responsibilities. Personal hygiene was a citizen’s duty, of benefit to the individual and the wider community (Seymour 2013). Ideas about good citizenship were entwined with moral pronouncements, something evident in interwar health education (Welshman 1997a). Hygienic citizenship was also especially prominent in a colonial context. Non-whites were often thought to have neither the civic status or responsibility necessary to be capable of achieving hygienic citizenship (Bashford 2003; Anderson 2006). Such beliefs persisted into the mid-20th and later 20th century, following colonial subjects to Britain through immigration. Migrants were expected to conform to their new home’s hygienic expectations, and they were often found wanting (Bivins 2015; Horton and Barker 2009). Efforts to inculcate hygienic practices in postwar Britain were not, however, confined to non-whites. Practising good hygiene to prevent infection was everybody’s business, but some people were thought to be more liable for this than others.

Responsibility for health, and whether this should be placed on individuals or the state, was a key element of other formulations of citizenship that began to move to the fore in postwar Britain. Chief among these was the concept of ‘social citizenship’. The origins of social citizenship can be traced to the late 19th century when ‘the social’ first emerged as a sphere of intervention for middle-class reformers (Oosterhuis and Huisman 2014). Improving social conditions and providing forms of social insurance, such as healthcare and pensions, were the basis of social citizenship, although this was often applied only to male workers. A more universal concept of social citizenship developed in the early to mid-20th century in tandem with the establishment of the welfare state and the growth of social medicine. In Britain and other parts of Europe, proponents of social medicine argued that social conditions influenced health, and the state should be responsible for addressing these through the provision of welfare. The establishment of the NHS in 1948 appeared to offer a kind of social citizenship based on collective rights. This was dictated by a social rather than purely an economic logic, one that justified interventionist planning by the state in order to achieve national efficiency (Porter 2011, 113). From the mid-1950s onwards, social medicine became less concerned with the impact of social structure on health, and more concerned with individual behaviour, resulting in an emphasis on responsibilities rather than rights (Porter 2002).

Despite the renewed focus on the individual social citizenship survived, and was remade in various ways as notions of both public health and the welfare state were reformulated (Taylor-Gooby 2009). One of the principle ways in which social citizenship was retained was through the encompassing of elements of consumer citizenship. At first this may seem to be an unlikely pairing, but in the early 20th century, consumer identity was tied to the development of welfare politics and social citizenship. Elements of this persisted into the middle of the century, as the ‘citizen consumer’ and the ‘rational consumer’ came into being (Trentmann 2006). By the 1960s, people were beginning to apply the principles of consumerism to public services, like healthcare. Consumer citizenship offered a way for patients and healthcare users to voice their own concerns around issues such as autonomy, representation, complaint, rights and information (Mold 2015). Of course, forms of health consumerism had long been in existence in Britain and elsewhere, including the purchasing of hygienic products and services (Tomes 1999; Smith 2008). Yet health consumerism was not solely about shopping or more choice for the individual, but could also be concerned with collective rights and responsibilities, a formulation that allied it closely to notions and practices of citizenship (Hilton 2009). There were many strands and variations of ideas about what it meant to be a good citizen. Hygienic, social and consumer citizenship, although distinctive, overlapped, intersected and sometimes conflicted. Considering the issue of food hygiene allows us to see how this played out in practice and begin to get to grips with its wider implications for citizenship and health in Britain in the mid-20th century.

## Food hygiene in postwar Britain

Food quantity and quality were a perennial concern for health authorities, but there were distinctive features about the postwar period in Britain that made food hygiene a public health issue of some significance. The impact of hunger and poor nutrition on health were well established by the late 19th and early 20th century, even if efforts to improve the situation were only partially successful (Webster 1982; Vernon 2007). As mass food production increased, so did the possibility that foods might be deliberately adulterated or accidentally contaminated. Regulations designed to improve food quality, especially essential products such as milk, were gradually introduced but food hygiene was difficult to control (Phillips and Smith 2013; French and Phillips 2000; Atkins 1992). In part, this was because food poisoning, although ever-present, was not defined until 1935, and not a notifiable condition until 1939 (Hardy 1999). Other factors also combined around this time to make food poisoning more visible and more prominent. Although rationing continued until 1953, there was a gradual return of consumer choice in foodstuffs and new, mass-produced foods were increasingly available (Oddy 2003). Changes in food production, manufacture and distribution meant that tainted foods, such as powdered egg, could be spread over a wide area. Mass catering during the war, and the increase in eating out immediately after it, also facilitated the occurrence of food poisoning on a large scale.

Indeed, there were a series of spectacular outbreaks of food poisoning in the immediate postwar period, such as incidences of typhoid in Halifax and Aberystwyth in 1946, and salmonella in two different hospitals in 1949 and 1950 (Hardy 2015, 82–83, 215). An outbreak of typhoid in 1964, traced back to contaminated Argentinian corned beef, left 500 people in Aberdeen in hospital and resulted in an official inquiry (Smith 2007; Smith et al. 2005). Over the period 1946–1965, it was estimated that there were nearly 100 000 cases of food poisoning (Smallman-Raynor and Cliff 2012, 148-9). Recorded incidences of food poisoning increased sharply during the 1950s, from 2428 per year in 1949 to a peak of 8961 in 1955 (Ministry of Health, 1965). Food poisoning became a notifiable condition in 1939, requiring those cases that came to the attention of the medical profession to be reported to the local Medical Officer of Health (MOH), but most incidents likely went undetected, meaning that the official number of cases was a significant underestimate. Although few episodes of food poisoning were fatal, they were nonetheless costly, resulting in absence from work and the use of NHS resources. Food poisoning thus represented an avoidable threat to both national productivity and the health of the community. The ‘productionist’ concerns about the strength and health of the workforce that had dominated the outlook of public health officials in the first part of the 20th century thus connected up with the more ‘communitarian’ ethos of the NHS and its emphasis on social solidarity (Pickstone 2000).

Other broader changes in the public’s health in the postwar period also helped to increase the attention given to food poisoning. As incidences of infectious diseases such as tuberculosis and diphtheria began to decline, food poisoning acquired greater significance as a microbial threat yet to be conquered (Barr 1978). The continued presence of food poisoning went against the grain of the changing pattern of disease, but chimed with the long-held perceptions and responsibilities of public health officials. Combatting infectious disease was something that MOHs in particular were comfortable with. Some historians have argued that MOHs continued to focus on infectious disease in the postwar period despite its declining importance and were unaware or unable to deal with new threats to public health, like smoking (Webster 1988, 377–78; Lewis 1986; Lewis 1991). Other researchers have called this into question, pointing to the enterprising activities of many MOH around such issues (Gorsky 2007; Diack and Smith 2002; Welshman 1997b). Irrespective of the individual MOH’s interests, compulsory notification and reporting of food poisoning meant that it was a problem that could not be ignored.

One way to combat this issue was through the introduction of legislation. In 1955, a new Food and Drug Act was passed. This imposed food hygiene regulations on establishments that provided food and the people that worked in them, such as the provision of hot water, nail brushes and towels. Legislation, however, could only go so far. As one Ministry of Health official commented in 1953 ‘new legislation cannot be effective unless there is a public opinion to demand and support it’ (Ministry of Health 1953). Another remarked that “Health education, in my opinion, is, to a great extent, the answer to this everyday problem” (Ministry of Health 1953a). From the late 1940s through to the end of the 1960s, a vast array of material, including posters, films, exhibitions and demonstrations on ‘clean food’ and the prevention of food poisoning were produced by the Ministry of Health, the Central Office of Information (COI) and local MOHs. The topic of food hygiene dominated public health education at the local level, forming a major part of exhibitions and other communication strategies employed by the MOHs (Mold 2018). Even by the mid-1960s, as other public health matters, such as the dangers posed by smoking, started to become more prominent, food poisoning was still a significant concern. In the first part of 1964, the Ministry of Health sent out 285 000 copies of posters on food hygiene, a number that easily eclipsed the 187 200 posters on smoking or the 36 000 on dental health that had been issued in the previous year (Ministry of Health 1964a). This level of activity can only partly be explained by the threat that food poisoning posed. Analysis of some of the materials produced points to a set of deeper issues.

## Public health information films

Visual and audio-visual sources produced as part of public health campaigns offer a uniquely valuable insight into the framing of and response to public health problems. The visual culture of public health in Europe and North America during the 19th and early 20th century has been explored by a variety of scholars, and attention is now shifting to the latter part of the 20th century as well as low-income and middle-income countries (Serlin 2010; Stein and Cooter 2011; Medcalf and Nunes 2018; Hand 2020). Analysing such material allows us to explore the complex relationship between the intentions of public health authorities, the media through which these were conveyed, their interpretation by a variety of audiences and the broader milieu in which such materials were located. Moving images, including documentaries and health education films, were thought to be an especially valuable part of the health educators’ arsenal. Health education films were made and shown in Britain from 1919 onwards, and there was a lively culture of municipal health cinema throughout the interwar period (Boon 2005; Lebas 2018). It is not surprising, therefore, that food poisoning was the topic of a series of public health information films made after World War II. In 1949, three films were made that were then shown to audiences throughout the 1950s and even in to the 1960s. There was a cartoon produced by the famous film makers Halas and Batchelor (who also made the ‘Charley’ series of films heralding the introduction of the NHS), entitled ‘A Fly About the House’ (Wells and Halas 2006, 26). Working with the Central Council for Health Education (CCHE) and the COI, the Ministry of Health also funded the film ‘Another Case of Poisoning’. In addition, the Ministry of Food commissioned ‘The Good Housewife in her Kitchen’, which was made by the COI. Alongside a set of instructions about the prevention of food poisoning, these films reflected and reinforced a set of wider tropes about gender, responsibility and citizenship.

Of the three films, only ‘Another Case of Poisoning’ survives intact and is easily accessible through the Wellcome Collection (Another Case of Poisoning 1949). ‘Another Case of Poisoning’ opens with a man in hospital suffering from food poisoning. He works in a food factory, a ‘very responsible job’ according to the doctor who is treating him. The source of the patient’s illness needs to be traced in order to prevent other people from experiencing a similar fate. The patient claims to follow all of the ‘rules and regulations’, but a flashback reveals that he does not, and neither, it seems, does anyone else involved in the handling of food. Various infractions are detailed, from the wife who takes the rubbish out and then touches fresh food without washing her hands, to the butcher with a dirty bandage on a cut finger and the barmaid who wipes a glass with a dirty rag. The man, it seems, could have picked up his case of food poisoning almost anywhere. Towards the end of the film, the doctor turns to the camera and tells the audience that food handlers must realise how responsible and important their jobs are. The health of every one of us, he says, depends on them. The doctor then outlines the steps that can be taken to prevent food poisoning, such as the regular washing of hands and covering food. The film concludes with another patient being admitted to the hospital, also with food poisoning. The doctor turns to camera once more and says: “I hope none of you were to blame”. Individuals, the film suggested, had a social responsibility to protect themselves and others from food poisoning.

In some ways, ‘Another Case of Poisoning’ was representative of a continuation of tropes seen in prewar health education films. As with the earlier films explored by Boon, the doctor was presented as the calm voice of authority, restoring order and dispensing good advice (Boon 2005). Like the prewar health education films ‘Another Case of Poisoning’ was also a moral tale, with the patient transgressing, and being punished for his (and everyone else’s) bad behaviour. There were also parallels with the more socially progressive municipal health films made by Bermondsey Borough Council which aimed to instruct citizens about their personal responsibility for health, as well as their entitlement to certain services (Lebas 2018). Where ‘Another Case of Poisoning’ differed was that such rights and responsibilities were now universal, available to everyone through the newly created NHS. This meant that the duties of social citizenship, in this case practising good food hygiene, were a responsibility for everyone, something reflected in the varied cast of characters depicted in ‘Another Case of Poisoning’.

A slightly different set of values and assumptions was on display in ‘The Good Housewife in her Kitchen’. The film began by instructing women how to store food in an ideal environment. This involved placing food in the refrigerator and using modern food storage materials, including plastic wrap. During the demonstration the female instructor is interrupted by one of the male film crew who tells her that his ‘missus’ does not work in such conditions. The instructor is transported to a set that looks more like a ‘real kitchen’, complete with range, open-shelved food storage and no refrigerator. Unfortunately, the final sections of the film have been lost, so we cannot know what advice was given to housewives labouring under such conditions, but these were certainly more common than the clean, bright modern kitchen shown in the first part of the film. For example, in 1951, only 56% of homes in Manchester had exclusive use of piped water, cooking stove, kitchen sink and water closet. Even by 1961, a fifth of houses in the city were without access to a hot water tap (Langhamer 2005, 350). In 1956, just 8% of British homes had a refrigerator, a figure that had only risen to 38% by 1964/5 (Rosen 2003, 14). This suggests that some of the guidance on food hygiene issued to housewives was out of line with the actual conditions under which most people lived. ‘The Housewife in her Kitchen’ shows that these were sometimes taken into account by public health authorities.

Such an appreciation of context, however, appears to have been the exception rather than the rule. By encouraging practices that many housewives may have found hard to achieve, there was a danger that food hygiene messages missed their target. In a more recent analysis of public health communication about a cholera epidemic in Venezuela in 1991, anthropologist Charles Briggs found that the use of images of women in well-equipped kitchens demonstrating cholera prevention measures was unsuccessful. Middle-class viewers thought such messages did not apply to them, and working-class viewers could not recognise themselves or the conditions in the images (Briggs 2003). It is difficult to know if the same is true of postwar food hygiene material, but widespread indifference towards these campaigns suggests that something similar may have taken place. Nonetheless, the Ministry view of housewives was that although they were in need of instruction, they were also able to act on these recommendations and become better hygienic citizens as a result.

Whether or not such change actually took place is hard to gauge. The trio of films made in 1949 had a fairly wide circulation over a long period. In 1953, the Ministry of Health estimated that over 750 000 people had seen ‘Another Case of Poisoning’, and the film was still being shown well into the 1960s (Ministry of Health 1953b). All three films were often shown together at local cinemas, on cinema vans which toured popular venues such as shopping centres, as well as at civic centres and other public places, like the baths (Medical Officer of Health for East Barnet 1950, 14; Medical Officer of Health for Dagenham 1960, 37; Medical Officer of Health for Camberwell 1950, 23). Some MOHs collected attendance figures for film showings or remarked in their annual reports on how popular (or not) they thought these had been. In 1950, the MOH for Camberwell held a showing of films including ‘The Good Housewife in the Kitchen’. Despite the fact that the showing was free, the MOH regretted to report that attendance was ‘poor, and one is compelled to conclude that this is due to lack of interest’ (Medical Officer of Health for Camberwell 1950, 23). Likewise, the MOH for Hayes and Harlington commented that at his film showing in 1951, ‘the response from the public was not good’, although ‘an attendance of more than 200 schoolchildren made the effort well worth while (Medical Officer of Health for Hayes and Harlington, 1951, 17)’. In contrast, the MOH for Willesden reported that in 1953 around 3500 people had seen films on clean food (Medical Officer of Health for Willesden 1953, 30). It is difficult to say what audiences made of these films, and even harder to assess whether or not people changed their behaviour as a result, but health educators certainly believed in the power of the moving image. In 1965, a Ministry of Health official proposed remaking ‘Another Case of Poisoning’ as though it was a ‘bestseller’ it was also ‘old-fashioned.’ The plan was stymied by the Treasury, but another official thought that an ‘outside organisation’ such as Heinz or Domestos might be encouraged to make a new film instead (Ministry of Health 1966). The production of health education films by commercial organisations was nothing new, gas and electricity companies had done so in the 1930s, but the move was representative of a broadening of the range of agencies thought to be responsible for public health (Boon 2008).

Around the same time, the burgeoning mass media, and especially the appearance of television in many people’s homes offered new opportunities for health educators to reach a wider audience (Berridge 2009). In 1963, the Ministry showed a series of brief ‘filmlets’ on food hygiene on both the BBC and commercial television. The filmlets were ‘often, but not exclusively, shown at lunchtime or in the afternoon when some housewives are looking in’, although ‘they also get shown at peak periods’ (Ministry of Health 1964c). The assumption that women were at home in the middle of the day and watching TV was potentially erroneous, as increasing numbers of women were working outside of the home in this period (McCarthy 2017). Nonetheless, women still shouldered the majority of domestic tasks and it was increasingly the case that middle-class as well as working-class women cooked and cleaned for themselves as the use of servants dwindled (Beaumont 2015, 168). Indeed, the focus of much food hygiene messaging on women and housewives was in-line with wider changes surrounding the position of women in society and their status as citizens. As Caitriona Beaumont has shown, women’s groups, such as the Women’s Institute, had put forward a notion of the housewife as a citizen since at least the 1920s, and this vision persisted and evolved in the postwar period (Beaumont 2015). Practising good hygiene as a part of being a good housewife thus tied in with wider perceptions of female citizenship. At the same time, ideas about the home were also shifting (Langhamer 2005). The home, and especially the working-class home, had long been of interest to public health authorities, but in the postwar period it was also increasingly the subject of social scientific research (Davies 1988; Lawrence 2013; Lawrence 2013; McCarthy 2016; Savage 2008). The home was a place where every day working-class lives could be examined, as could understandings of related concepts like class and community. All of this combined to make the home a continued place of importance for health authorities and one of the ways into the home (literally and metaphorically) was through food hygiene. Public health information films on food hygiene, through their depiction of homes and, by the 1960s, their movement into the home itself through television helped reinforce dominant understandings of the home and the housewife, and hinted at a new emphasis on collective and individual responsibilities for public health.

## Public health posters

This combination of older ways of thinking about public health issues with more novel dimensions can also be observed in some of the posters produced to educate the public about food hygiene. Indeed, the very design of such material embodied the melding of old and new, where up-to-the minute graphical approaches were often used to depict ancient foes. Between the late 1940s and the late 1960s, the Ministry of Health and the Scottish Home and Health Department produced a large range of different posters on the topic. It is impossible to attempt a comprehensive analysis of all of these here, but viewing these as a corpus, and homing in on a few examples in more detail, allows for the delineation of certain themes. Many posters made a connection between cleanliness and modernity, something further underscored by the pictorial and graphical modes employed. The pairing of cleanliness and modernity was nothing new, but during the 1950s and early 1960s, as David Kynaston suggests, ‘modernity’ was the ‘spirit of the age, epitomised by the desire in relation to the built environment to dump the past, get up to date and embrace a gleaming, functional, progressive future ’ (Kynaston 2015, 46). ‘Modernity Britain’ had many manifestations, such as the clean lines of New Towns; the cleansed environments of redeveloped urban spaces and cleaner homes heated by gas or electricity rather than coal (Clapson 1998; Smith 2019). Environmental measures, such as the burning of smokeless fuel and the reduction in smog following the introduction of the Clean Air Act in 1953, also helped to heighten perceptions of the importance of cleanliness (Jackson 2005; Nead 2017). Observers pointed to the growth of a ‘clean’ and ‘bright’ style that was popularised by the Festival of Britain in 1951 (Atkinson 2012; Conekin 2003; Hopkins 1963). This attention to cleanliness may have contributed to heightened interest in food hygiene, as members of the public supposedly felt that eating establishments should ‘look clean’ (Barr 1978).

The appearance of cleanliness and the use of visual methods to communicate this filtered through into food hygiene posters. Prewar hygienic messaging, as James Stark and Catherine Stones note, tended to cling to notions of miasma and contagion in the anthropomorphic representation of ‘germs’ (Stark and Stones 2019). In contrast, the food hygiene posters from the 1950s and 1960s focused more directly on how food became contaminated and what could be done to prevent this. The posters were striking and made use of techniques, such as mixing photographic images in black and white with large blocks of colour, that point to a novel, modern aesthetic. One poster was produced by the famous graphic designer Reginald Mount, but most of the posters are unsigned and there is little information in the archives about who designed the posters, or what their exact brief was. Yet the design of the posters was clearly important to officials. Indeed, one wondered “if we are providing ‘good’ design at the expense of punch and action-getting?” (Ministry of Health 1964b) There was a feeling among some Ministry of Health civil servants that “our poster designs on food hygiene are rather above the level of appreciation (and action) by the generality of food handlers” (Ministry of Health 1964d). Following a meeting with the COI, the same official remarked that there was a need to ‘produce simple uncluttered designs for simple cluttered people handling food’ (Ministry of Health 1964e). The assumption that food handlers were ‘simple’ and ‘cluttered’ may have been confined to one official, but it hints at a more general attitude towards the creation of hygienic citizens. Officials believed that food handlers were capable of absorbing messages, but these needed to be conveyed in the simplest way possible. This can also be seen in attempts to create posters that could be read by ‘foreign’ workers. The Ministry considered producing posters with no words but decided that ‘a pictorial poster cannot show the how/when/where/why/which (as appropriate) is an essential part of our message’. Instead, the Ministry plumped for the production of generic posters with space for overwriting in a variety of languages including ‘Pakistani; Hindustani; Urdu; Spanish; Italian and Greek’. There was a recognition that such posters needed to be displayed alongside English language versions, as ‘if only the foreign language were given and there were a mixed staff of English-speaking and non-English-speaking food handlers, there would be a feeling of discrimination on the part of the foreigners’ (Ministry of Health 1964f). Ministry officials seemed to have had a fairly low opinion of food handlers and their ability to act on food hygiene messages. Nonetheless, they attempted to make them into hygienic citizens by provoking behaviour change.

The behaviours the posters were intended to instil concentrated on a set of preventive strategies. These included the need to wash the hands and cover cuts when preparing food; how to wash up dishes and utensils; how to cool and store food; not to smoke at food counters as well as keeping flies off food. Most posters were explicit that the measures they were recommending were designed to prevent food poisoning, although this was sometimes framed under the heading that one should follow instruction ‘For health’s sake’. Indeed, beneath the specific images and words used in the posters other messages were being communicated that went beyond the prevention of food poisoning. Deep-seated anxieties and long-running tropes can be detected in the images used in some of the posters. There was a particular fascination with the vectors of transmission: be they zoonotic (rats and flies); inanimate objects (bins, utensils and crockery) or human body parts (especially the hands). Rats, of course, were an ancient enemy for public health authorities and they often figured centrally in the public health exhibitions mounted by MOH (Hardy 2019). Flies also had a long association with dirt and disease and stopping flies from carrying ‘germs’ which ‘infect uncovered food and cause food poisoning’ was a common instruction (Rogers 1989; 1989b). Insect vectors, at home and abroad, had featured heavily in wartime health messaging. The horrified fascination with flies persisted. One poster, produced in 1961, featured a magnified photographic image of a fly and the command to ‘Guard food against flies’. The oversized fly with its grossly magnified body, wings, spindly legs and beady eyes is clearly intended to provoke disgust, even fear. The black fly contrasts with the bright, clean block of colour in the rest of the poster and the criss-cross grid reminiscent of a net or mesh intended to prevent flies from landing on food. The text, with its use of the terms ‘guard’ and ‘germs’ gestures towards the martial language employed in prewar food hygiene posters, but re-worked for a modern age.

Indeed, the posters reflected a set of social and economic changes that widened the range of settings where food hygiene messaging was thought to be particularly apt. A number of posters and leaflets concerned food hygiene while on holiday. Two posters, produced by the COI for the Scottish Home and Health Department in the mid-1960s, depicted a family consisting of mum, dad, son and daughter at the beach. In one image, they are heading to the beach for a picnic, with the instruction to ‘Wash your hands before eating’ and ‘Holiday health depends on holiday hygiene’. Alongside a celebration of the virtues of the white, nuclear family and the affluence required to be able to take a holiday, other practices were being extolled. The poster is reproduced in a collection curated by Hester Vaizey, and in her note on this image she points out the increased entitlement to paid holiday for British workers was accompanied by a responsibility to ‘return to work healthy and refreshed, and the promotion of hygienic practices went a long way to ensure that’ (Vaizey 2014). Old concerns were in operation in new settings: food hygiene offered a way to reference and reinforce long-running moral codes and apply these to the novel scenarios of postwar Britain. Thus, the creation of hygienic citizens was, at least in part, a moral project.

## Codes of conduct

The moral dimension to food hygiene messages was most prominently on display in codes of conduct and other more direct modes of instructing the public in good practice. Responsibility for food poisoning was often linked to conduct. ‘Habits’, both ‘good’ and ‘bad’, as well as ‘standards’ and ‘manners’, pepper official correspondence on the topic. In 1956, Pat Hornsby-Smith, Parliamentary Secretary to the Ministry of Health, told an audience at the Greater London Rally of the Women’s Auxiliary Leagues of the Licenced Trade that ‘The aim of the new Food Hygiene Regulations [passed in the wake of the 1955 Food and Drug Act] has been to set a workable and enforceable common sense standard of good manners in food handling’. Although these regulations were expressed in legal terms, “they boil down to asking for the same cleanliness, care, and the same good manners in the handling of food as you would expect in your own homes”. The mobilisation of ‘common sense’ was a broader trope employed by a variety of bodies (both within health authorities and in other spheres, like the media) in relation to various public health issues, such as polio vaccination (Millward 2017). In this instance, implying that food hygiene was ‘common sense’ suggested that there was nothing new about such regulations, or that these were beyond the reach of the common (wo)man.

The actions hygienic citizens were expected to perform in order to prevent food poisoning involved engaging in certain practices and desisting from others. Many of these involved ‘common-sense’ behaviours such as washing hands, crockery and utensils; storing food correctly; eliminating pests and disposing of waste. Such practices may have been difficult to achieve for some working-class women, especially those without ready access to hot, running water or cold storage. But in their appeal to behaviour change, food hygiene campaigns were reflective of both a long-running concern with working-class ‘habits’ on the part of middle-class reformers and a new, more focused interest in individuals and their lifestyles. Personal habits, especially those around cleanliness and bodily hygiene, were of interest to late Victorian and early Edwardian public health authorities (Crook 2016). In the postwar period, the focus on what the epidemiologist Jerry Morris described as ‘ways of living’, or ‘mass habits and social customs’ expanded and intensified as these became more strongly connected to disease and ill-health (Morris 1955). From the 1950s onwards, a series of behaviours, including diet, exercise, smoking and alcohol consumption were linked to public health problems like heart disease and cancer. As the importance of individual behaviour as both cause and remedy for public health problems grew, its meaning altered. Sociologist David Armstrong argues that behaviour was no longer understood to be either the product of biological characteristics, or of habits, but of less deterministic forces located in a sense of individual identity and agency (Armstrong 2009). The reconfiguration of behaviour was part of a wider reimaging of the ‘self’. Through the ‘psy’ sciences the interior and exterior world of individuals was re-cast, as conduct became a project of self-government (Rose 2010; 1999). Behaviour change was central to the production of new, modern, kinds of selves (Vernon 1997; Jackson and Moore 2020).

The types of behaviours these new selves were expected to engage in included hygienic practices like washing hands, but these also overlapped and intersected with other seemingly desirable behaviours. This can be seen in an ‘information note’ on food hygiene issued by the Ministry of Health in the summer of 1964. The document stated that ‘Clean hands will not spread any infection. Hand-washing with soap and water after using the WC and before and after preparing different food items should be a normal everyday habit in the home’. In addition to such advice, the note also provided a wider set of rules that went beyond how to store and prepare food. The document asserted that ‘Protection of the family lies in personal hygiene, kitchen hygiene and good management in buying, storing, cooking and cooling the food’. Housewives should ‘influence traders by making it clear that they choose those shops and premises which take special care to ensure the freshness, cleanliness and good storage of foods they sell’(Ministry of Health 1964f). A similar approach was put forward in the Ministry of Health’s instructions to the Federation of Women’s Institutes in 1965. The Ministry asserted that women could ‘encourage good food traders and caterers by praising them and removing their custom from those whose standards are low’ (Ministry of Health 1965c). In a 10-point food hygiene code for housewives, produced by the Ministry of Health and the CCHE in 1967, women were instructed to ‘Buy only from clean places’ (Ministry of Health 1967c). The guidance suggested that the good housewife should keep herself and her kitchen clean, and should plan her purchases carefully and buy food only from ‘clean’ shops. The power of women as consumers, as ‘super-shoppers’, was acquiring greater weight at this time, and here it was being marshalled towards the improvement of standards in shops as well as in the quality of goods (Hilton 2003; 2002). Being a good hygienic citizen involved cleanliness and wise shopping. Notions of hygienic citizenship overlapped and intersected with ideas about consumer citizenship, helping to produce a new ideal type of citizen.

The success or otherwise of such projects can be called into question. The public response to food hygiene messaging is difficult to gauge. It is hard to know how many people saw food hygiene posters, films and exhibitions, and even more difficult to know what they made of them. Given the number of posters and leaflets produced, food hygiene messages must have been hard to miss. Whether people took any notice of them, however, was much more debateable. After receiving food hygiene publicity material, the MOH for Staines wrote to the Ministry in 1964 stating that “I feel I must say that while this material has some value, the impact on the public is so small as to be negligible” (Ministry of Health 1964h). A Ministry official replied: “I agree with you that it is difficult to get much impact on the public in the matter of food hygiene. Educating people and changing their habits is unfortunately a slow business which takes much time” (Ministry of Health 1964i). In 1966, the Food Hygiene Advisory Council reported that

there has been of late some improvement in the public’s awareness of the need for good food hygiene. Public apathy, however, continues to be the greatest single obstacle to the long term success of food hygiene education. Basic hygiene principles are accepted as sound, but there is widespread reluctance to put them into practice, and it is against this background that publicity efforts must be seen. (Ministry of Health 1966a)

Nonetheless, incidences of food poisoning declined from a high point in the mid-1950s, something the Council attributed to better practices among the public and food handlers following the introduction of stricter regulations in the wake of the 1955 Food and Drug Act. Other official missives were more willing to point to health education as the reason for improvements, but is hard to make an accurate assessment of the extent to which such materials led to behaviour changes (Ministry of Health 1964g). Indeed, the effectiveness of health education in changing individual behaviour was later called into question in other spheres, including antismoking and anti-alcohol campaigns, and efforts to prevent coronary heart disease (Berridge and Loughlin 2005; Frankel, Davison, and Smith 1991; Mold 2017). However, the question of whether or not health education could alter behaviour is somewhat beside the point. Food hygiene campaigns reveal a set of deeper concerns about the conduct of individuals and what it meant to be a good citizen that were both of their time and echoed longer-running issues.

## Conclusion

**Figure 1:**
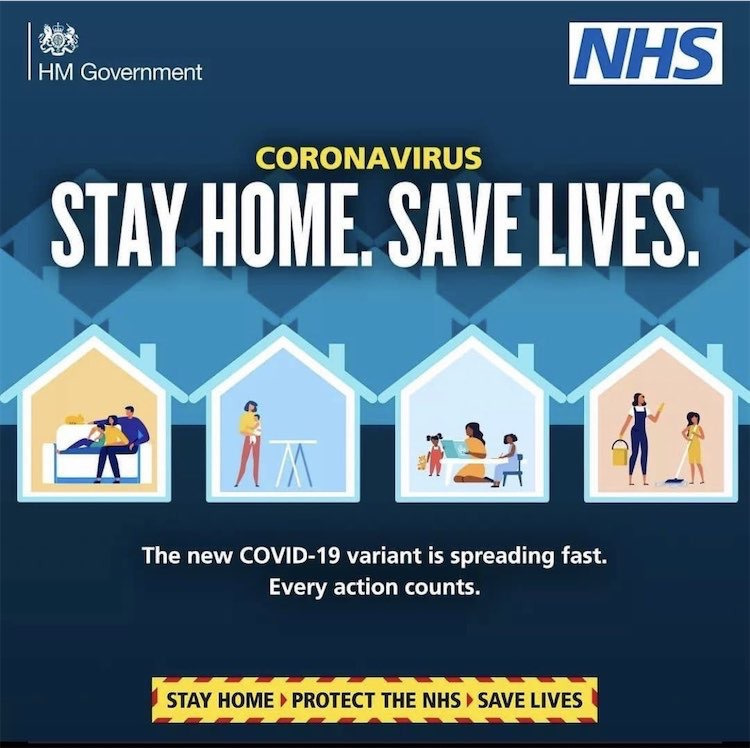
Stay Home, Save Lives. HM Government/NHS, 2021. This figure is covered by the Creative Commons Attribution 4.0 International License. Reproduced with permission of Crown; copyright Crown, all rights reserved. This information is licensed under the Open Government Licence V.3.0. NHS, National Health Service. To view this licence, visit http://www.nationalarchives.gov.uk/doc/opengovernment-licence/

In the spring of 2020, hygienic citizenship made a dramatic move to the forefront of contemporary public health policy and practice in Britain and elsewhere. In the early days of the COVID-19 pandemic, handwashing and other hygienic practices were presented as a way for citizens to protect themselves and others. Such campaigns were clearly a manifestation of hygienic citizenship, but they also encapsulated the other kinds of citizenship pointed to by this article. A form of social citizenship that emphasised collective responsibilities underpinned public health recommendations from handwashing to staying at home. Although it is questionable whether panic buying alcohol hand gel or antibacterial soap can be considered as a form of ‘citizenship’, it was certainly the act of citizens behaving as consumers. Hygienic concerns overlapped and intersected with social responsibilities and consumer demands. At the same time, old tropes continued to be recycled. In January 2021, one image produced as part of a government campaign designed to encourage people to stay at home made a brief appearance before being withdrawn (figure 1). The image depicted four homes in cross-section. In three of the four homes, women were engaged in childcare and housework. In the remaining home, the only one depicting a man, a family were shown relaxing on a sofa. When numerous commentators pointed to the gendered and heteronormative assumptions at work in the image, it was swiftly deleted, but the fact that such an image was designed, produced and put into circulation speaks of the longevity of gender inequality, and the ways in which public health messaging allows these to be voiced. Hygienic citizenship as a way of constructing the public, the management of its health and its relationship with government, cannot be washed away.

The enduring power of hygienic citizenship should come as no surprise. As I have suggested in this article, efforts to promote good hygiene and prevent food poisoning served as a vehicle for the expression of deep-seated fears and the voicing of novel concerns. Long-running connections between hygiene and morality, the behaviour of specific population groups, like women and the management of certain spaces, such as the home, were vividly on display in food hygiene publicity. These intersected with novel issues, such as new concepts of modernity and cleanliness, the changing position of women in society and the growth of consumerism as well as a renewed interested in the behaviour of individuals as both cause and remedy for public health problems. This resulted in the production of a new ideal of public health citizenship, a conception that melded hygienic practices with elements of social and consumer citizenship. The success or otherwise of this project can be called into question. Public health officials were sceptical about the extent to which citizens took their advice on board and acted accordingly. Getting individuals to change their behaviour through health education was difficult. Nonetheless, it became a key part of public health policy and practice as ‘lifestyle’ and individual behaviour were regarded as the primary causes of public health problems. Although there was some recognition that behaviour was shaped by context, that not all individuals operated in the conditions that allowed for the best hygienic practices, most emphasis was placed on what the individual should do to guarantee their health and that of those around them. Some individuals (especially women) were seen as being more responsible than others, but there was a growing sense of universalism in health education messaging. In this way, food hygiene campaigns prefigured the general direction of public health policy and practice from the 1960s onwards. Health was increasingly an individual responsibility as well as a social right.

Yet, as the COVID-19 crisis reminds us, the construction of healthy citizens is far from a unidirectional process. The making of healthy citizens was something asserted from above: health citizenship was a practice as well as a status, one that could invert the very rules on which it was founded. Resistance, outright rejection or apathy in the face of public health campaigns was representative not necessarily of a ‘failure’ of the tactics employed but of a wider gap between public health and its publics. Citizenship narratives could offer a way to lay claims and to resist and rebel. Perhaps there never was, never could be, or even can be, a perfectly healthy citizen, but in their making and unmaking we can see the complexities of the fragile relationships between government and the governed.

## Data Availability

Data sharing not applicable as no datasets generated and/or analysed for this study. N/A.
